# Effect of Self-Made TiO_2_ Nanoparticle Size on the Performance of the PVDF Composite Membrane in MBR for Landfill Leachate Treatment

**DOI:** 10.3390/membranes12020216

**Published:** 2022-02-13

**Authors:** Huiya Wang, Keqiang Ding

**Affiliations:** School of Environmental Engineering, Nanjing Institute of Technology, Nanjing 211167, China; whyplgl@njit.edu.cn

**Keywords:** landfill leachate, self-made TiO_2_, ultrafiltration membranes, MBR, PVDF, nanoparticle, composite membrane

## Abstract

The pollutant composition of landfill leachate is complex, and pollutant concentrations change greatly. Moreover, landfill leachates can easily penetrate into the soil and eventually pollute the ground water, which can cause environmental pollution and threaten human health. At present, landfill leachate treatment technology is still not mature. In this paper, the A/O-MBR (Anoxic–Aerobic Membrane Bioreactor) process is proposed to treat landfill leachate. To increase the hydrophilicity of the membranes and reduce the pollution of the membranes, the self-made TiO_2_ nanoparticles were used to modify the ultrafiltration membranes (PVDF-2). Meanwhile, PVDF-2 composite membranes showed the best separation performance. The optimum operating parameters were determined by changing the concentration of the pollutants in the reactor and selecting the dissolved oxygen, pH, and hydraulic residence time. The results show that the optimum operating conditions of MBR are mixed liquor suspended solids (MLSS) = 3200 mg/L, DO = 1.5–2.5 mg/L in a nitrifying tank, DO = 0–0.5 mg/L in a denitrifying tank, pH = 7–8, and a hydraulic retention time (HRT) = 5 h. To reach the “Discharge Standard of Pollutants for Municipal Wastewater Treatment Plants” (GB18918-2002), the effluent of the MBR system further enters into the RO system. This work presents an environmentally friendly synthesis of TiO_2_ nanoparticles and added into PVDF. The addition of self-made TiO_2_ in PVDF membrane has improved the antifouling performance significantly, which has the potential for the treatment of landfill leachate.

## 1. Introduction

With the acceleration of urbanization, the amount of domestic waste produced by residents has increased significantly. Solid waste treatment is usually used to treat landfill sites because of its low treatment cost and low technical requirements [1,2,3,4,5]. However, solid waste treatment will lead to the generation of landfill leachate. The treatment of landfill leachate has become a hot issue in the field of environmental protection and water treatment. Moreover, the stricter pollutant discharge standards put forward higher requirements for landfill leachate treatment technology [6,7,8,9].

Landfill leachate has the characteristics of an extremely complex organic pollutant composition, large fluctuations in water quantity and water quality, an imbalance of its nutrient element ratio, and a large number of toxic and harmful substances [10,11,12]. Therefore, it is difficult to deal with landfill leachate sufficiently by relying on the conventional sewage treatment process [13,14,15,16]. At present, landfill leachate treatment processes around the world include physical and chemical treatments, biological treatments, ecological treatments, anaerobic ammonia oxidation, and other operational processes [17,18,19,20,21,22,23,24,25]. Physicochemical methods mainly use physical and chemical reactions to degrade and remove pollutants in landfill leachate; these methods include flocculation and sedimentation, physical adsorption, blow off, membrane filtration, and advanced oxidation technology. Biological methods have the advantages of less economic investment, high treatment efficiency, and stable operation and have been widely used around the world. The state treatment method is a method that uses the ecological cycle of carbon, nitrogen, phosphorus, and other elements in the ecological environment to treat landfill leachate with the joint action of microorganisms and plants in the soil. The ecological treatment method has the advantages of simple operation and management and a low operational cost but also has the disadvantages of a large floor area, low treatment load, low treatment efficiency, etc. With an increase in the treatment capacity and ecological elements in the system, the concentration of heavy metal salts and refractory pollutants in the soil will gradually increase, which will affect the growth of plants. If the constructed wetland is not well closed, it may invite pollution into the surrounding environment and groundwater. It is difficult for traditional biological treatment processes to meet the new discharge standards for landfill leachate, and there is an urgent need for new landfill leachate treatment technology. In recent years, researchers have done a lot of research on the treatment technology of landfill leachate. For example, Wang et al. [26] used the UASB-MBR process to treat landfill leachate. The results showed that the COD of influent water was 1491–2965 mg/L, the ammonia nitrogen of influent water was 642–980 mg/L, the COD and ammonia nitrogen of effluent water were 588 and 323 mg/L. Compared with them, the pollutant concentration of influent water was smaller, the COD removal rate was 17.84% higher than them, and the ammonia nitrogen removal rate of this study was 4.69% higher than them. Wang et al. [27] used iron carbon micro-electrolysis to treat landfill leachate. The results showed that the removal rate of COD and ammonia nitrogen decreased by 15% and 18%, respectively. The advantage of this process is that there is no excess sludge, but the removal rate of this process is relatively low. E. Diamadopoulos et al. [28] adopted the method of reinjection to treat the landfill leachate. The removal rate of COD and BOD is more than 90%. However, at present, the reinjection treatment cannot completely eliminate the landfill leachate, and would produce odor and other polluting gases, resulting in secondary pollution. In addition, Li et al. [29] used the electrochemical oxidation method for landfill leachate treatment, the removal rate of COD was 90.16%, and the removal rate of ammonia nitrogen was 100%, but the influent COD of this process and ammonia nitrogen is relatively low (only 693 and 263 mg/L). This method is only applicable to the landfill leachate with low concentration influent water. Li et al. [30] adopted an anaerobic–aerobic biological fluidized bed to treat the leachate. The concentration of influent COD and ammonia nitrogen was 5000 and 280 mg/L but the effect of effluent water is not ideal.

The membrane bioreactor (MBR) is a new technology combining biological treatment and physical separation, which can effectively replace the traditional secondary sedimentation tank separation [31,32,33]. Membrane bioreactors (MBRs) combine biological and membrane separation processes and have been widely applied to industrial wastewater, enabling its reuse [34]. Over the last two decades, MBR reached a significant market share in the wastewater treatment business, and it is expected to grow at a compound annual growth rate of 13.2%. Compared to conventional activated sludge systems, MBRs have a low area demand (2–10 times lower), low sludge production (2 times lower), higher sludge retention time and total removal of suspended solids, high removal efficiency of micropollutants and persistent organic pollutants, and low sensitivity to wastewater composition. Compared with the traditional activated sludge process, the application of MBR in landfill leachate treatment has great advantages, mainly because MBR greatly reduces sludge and its hydraulic retention time allows biological treatment to maintain a high sludge concentration and produce less excess sludge. At the same time, it can make the effluent water quality more stable and energy consumption lower. Compared with the traditional activated sludge process, the MBR process has the following advantages: 1. The mud water separation efficiency is high, and the filtration and separation efficiency of microbial particles or suspended solids and polymer organic matter is as high as 100%. 2. No loss of microbial flora. When biological methods are used to treat sewage, the biochemical system can cultivate bacteria with special degradation ability for specific pollutants, which will not be reduced. The high concentration of microorganisms in the reactor can promote the degradation of organic matter. 3. In the MBR process, the sludge residence time (SRT) in the reaction tank is long, which is conducive to the proliferation of slow-growing bacteria and improves the degradation ability of microorganisms to pollutants. 4. The MBR process has low excess sludge output, long sludge residence time, and stable sludge properties. 5. In the MBR process, because membrane separation has a good interception effect on microorganisms and macromolecular substances, the effluent is stable, the biochemical system can maintain high microbial concentration, high treatment load, and a good pollutant removal effect. 6 Compared with secondary sedimentation tanks, a MBR membrane separation system has the advantages of compact equipment (greatly reduced floor area), simple operation of membrane separation components, remote automatic control, and so on. The MBR process is used to treat landfill leachate with large fluctuations in water quality and quantity. The effluent quality is stable, and the system has strong load impact resistance [35].

Combining the properties of nanoparticles and polymers, PVDF doped with nanoparticles can have damaged structural symmetry to induce the formation of β-phas. For example, PVDF-TiO_2_ membranes have been extensively studied to achieve improved antifouling capability for the separation of oil–water mixtures, organic matter, and proteins [36,37,38]. These reported PVDF-TiO_2_ membranes were prepared via either blending or post-coating. However, the blending method has difficulties in obtaining high exposure of TiO_2_ on the membrane surfaces, and the post-coating approach could not deliver conformal and durable coatings, especially on the internal pore surfaces of membranes with relatively small pore sizes, which could limit the performance improvement from TiO_2_ addition. Therefore, there is a need to develop an in situ TiO_2_ coating method that is capable of achieving conformal and durable TiO_2_ coatings on both the top surface and internal pore surfaces of the PVDF membrane. A membrane bioreactor is mainly determined by the membrane. The membrane is easily contaminated, which leads to a decrease in flux and the need for frequent cleaning and replacement, leading to an increase in operational costs. This paper presents the results of different TiO_2_ effects on antifouling properties of TiO_2_/PVDF nanocomposite membranes in an MBR system in order to treat the landfill leachate. Notably, the TiO_2_ was made by us, which clearly increased the mass transfer rate of the solvent and non-solvent during the process of membrane formation, which is conducive to the formation of pores and increasing the roughness of the membrane surface, thus effectively improving the water flux of the membrane. Moreover, the TiO_2_ nanoparticles are self-made, which makes them more conducive to dispersion in the casting solution compared to commercial options. In the leachate experiment, the 5% TiO_2_/PVDF has the best effect on the treatment of landfill leachate and achieved average removal rates of COD, ammonia, and total nitrogen of 87.84%, 92.97%, and 89.95%, respectively.

## 2. Materials and Methods

### 2.1. Sample Preparation

#### 2.1.1. Preparation of TiO_2_

**Figure 1:**
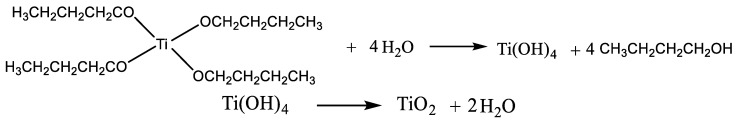
Formation process of TiO_2_.

The TiO_2_ was prepared by sol-gel process (Figure 1). Specifically, 10 mL tetrabutyl titanate and 35 mL absolute ethanol were added into a 250 mL beaker, then the beaker was placed in a magnetic heating mixer and stirred for 30 min to obtain solution A at room temperature. Then, 4 mL of glacial acetic acid were dropped into 10 mL of deionized water, then anhydrous ethanol and HCl were added to adjust the pH to less than 3 to obtain solution B. Solution A was added to solution B slowly through a constant pressure funnel at a rate of 3 mL/min to obtain a yellow solution. Then, it was placed in a magnetic heating mixer for stirring for 2 h at 40 °C. The obtained solution was put into a vacuum drying box and dried for 24 h at 80 °C vacuum to obtain a light yellow crystal. The light yellow crystal was put into the box type resistance furnace for heat treatment at 500 °C for 2 h and cooled to normal temperature. The obtained crystal was ground to obtain white titanium dioxide particles.

#### 2.1.2. Preparation of PVDF-X

All the membranes used in this experiment were prepared by the phase inversion method (the polymer solution was immersed in a non-solvent bath and the polymer precipitated rapidly at the interface leading to the formation of a very thin dense layer and a porous layer under the dense layer). Firstly, poly(vinylidene fluoride) (PVDF, 50–70 million) powder, two glass plates, and two glass rods were put into an oven and dried at 50 °C until the PVDF reached a constant weight. Secondly, 21 mL N-N dimethylformamide (99.5%) were added into the TiO_2_. Then, the PVDF was dissolved by an ultrasonic wave and stirred using a magnetic stirrer for 2 h at 60 °C. After the PVDF powder was completely dissolved, the glass plate and glass rod were removed from the oven, and the cast film was taken out while hot. The liquid was placed on the glass plate while rolling the glass rod so that the casting membrane liquid was evenly coated on the glass plate; then, the glass plate was put into deionized water, and the film falling off the glass plate was washed and dried. Without adding particles was recorded as PVDF-0, commercial TiO_2_ was recorded as PVDF-1, and self-made TiO_2_ was recorded as PVDF-2.

### 2.2. Characterization of Samples

The morphology was observed with a JSM6380LV scanning electron microscope (SEM). Fourier transform infrared spectroscopy (FT-IR) was performed using a NEXUS 970 Fourier transform infrared spectrometer from NICOLET (Company of the United States). The thermogravimetric analysis (TGA) was performed on a TG209 (Netzsch Co. Germany). The contact angle was measured by a contact angle measuring instrument (Kruss, DSA30, Germany). The phase separation rate of casting solution was completed using a TU-1901 ultraviolet spectrophotometer manufactured by Beijing pugetong Instrument Co., Ltd. (Beijing, China) The porosity of the membranes was determined by the dry–wet membrane gravimetric method.

### 2.3. MBR and RO Setup and Operation

**Scheme 1:**
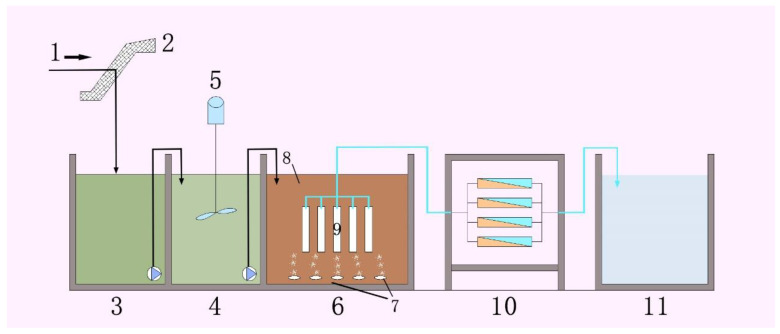
Experimental device schematic. 1. Inlet. 2. Grille. 3. Regulating pool. 4. Anaerobic pool. 5. Anaerobic device. 6. The MBR pool. 7. Aerating apparatus. 8. Activated sludge. 9. Membrane. 10. Reverse osmosis unit. 11. Clean-water reservoir.

The designed reactor consisted of anoxic denitrification and aerobic nitrification sections. Firstly, the inlet water entered the denitrification tank and then entered the nitrification tank through internal circulation. The MBR membrane module adopted a built-in MBR, and the ultrafiltration membrane module was placed in the aerobic aeration tank. The aeration head was used to prevent the microorganisms from adhering to the ultrafiltration membrane, which prevented and reduced membrane pollution. PVDF flat film was used in the membrane module of the A/O-MBR system. The designed device is shown in Scheme 1. Specifically, the device was divided into two tanks: one was an aerobic tank, which used aeration to increase the dissolved oxygen in the water, and the other was an outlet tank, which used a water pump to pump water out of the membrane module and used a flowmeter to adjust the hydraulic retention time. In addition, the anaerobic tank used a bucket with a smaller mouth than the other tank. The shape of the reaction tank was the same; the size was L × W × H = 33 × 30 × 37 cm^3^, the effective volume was 36 L, and the inflow flow was 15 L/h.

The membrane module adopted an intermittent suction type water outlet mode (operated for 10 min and then paused for 2 min), the aeration mode used aeration head aeration, the reactor temperature mainly changed at 15~32 °C (without manual control), and the water temperature during stable operation was 28 °C. During the experiment, the reactor discharged sludge in the form of the sampled and natural sludge discharge. The sludge mass concentration in the MBR was maintained at 3200 mg/L, DO = 1.5–2.5 mg/L in the aerobic tank and DO = 0–0.5 mg/L in the anaerobic tank, pH = 7.0–8.0, and HRT = 4 h. During the experiment, the operational mode of the A/O-MBR reactor was as follows (Table 1):

(1) The effluent peristaltic pump was operated intermittently for 10 min and stopped for two minutes;
(2) The leachate in the anaerobic tank entered the aerobic tank of the A/O-MBR reactor through siphonage;
(3) During the experiment, DO, HRT, pH, and the other parameters in the system were strictly controlled, and the content of COD, ammonia nitrogen, and total nitrogen in the inlet and outlet water of the system was monitored in real time.

The effluent of the MBR system entered into the RO system under the pressure of a special water pump. The concentration separation and interception were completed and the refractory organics and part of ammonia nitrogen were separated. The separation concentrate was partially recovered at the outlet. At the same time, it was able to desalt the effluent after MBR treatment, so as to ensure that the effluent quality after the subsequent treatment can reach the standard. The nanofiltration was connected in series in the overall structure combination construction. In this process, the concentrated liquid produced by RO system can be collected and recycled in the A/O process. The device ran for about 19 h every day, and the daily processing capacity was about 285 L/d.

### 2.4. Pure Water Flux Measurement

The pure water flux of the membrane was measured by a cup ultrafilter. Specific steps: Under certain operating conditions, the defect-free modified membrane was first cut into a disk with an effective membrane area of 0.0047776 m^2^ and a diameter of 7.8 cm. Then, the disk was placed in an ultrafiltration cup and the pure water flux of the membrane was measured at a nitrogen pressure of 0.1 MPa for a certain period of time. All membranes were measured in parallel three times to eliminate errors. The pure water flux was calculated according to Equation (1):

(1) $$J_{0}=\frac{\mathrm{Vw}}{\mathrm{At}}$$

V_W_ (LMH) is the volume of pure water passing through the membrane, A (m^2^) is the area of the membrane to be measured, and t (h) is the test time.

### 2.5. Bovine Serum Albumin (BSA) Retention Rate

**Scheme 2:**
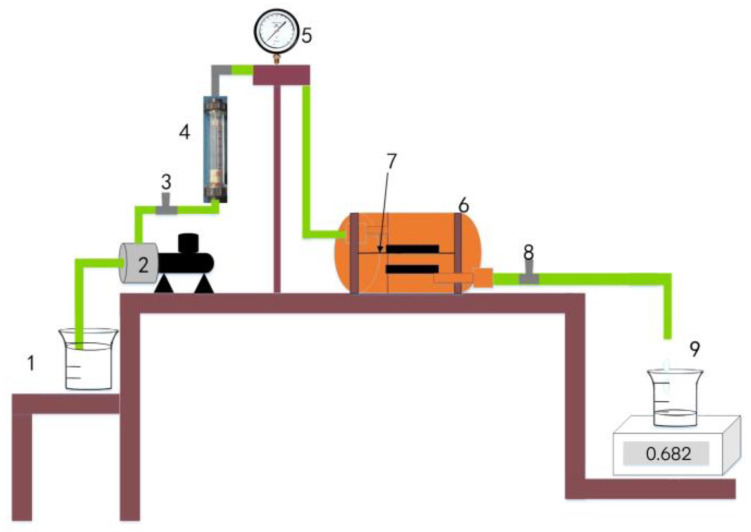
Membrane separation equipment. 1: Container for solution; 2: Pump; 3: Inlet control valve; 4: Liquid flow meter; 5: Inlet pressure gauge; 6: Flat membrane module; 7: Flat sheet membrane; 8: Outlet control valve; 9: Permeation.

At room temperature and pressure of 0.1 MPa, the PVDF composite membrane was used to separate the BSA solution (Mn = 67,000) with a mass concentration of 1 g/L through a glass sand core filtration device (as shown below), so as to determine the rejection rate of the membrane (Scheme 2). The calculation formula was as shown in Equation (2):

(2) $$R=(1-\frac{C_{1}}{C_{2}})\times 100\%$$

R is the retention rate; C_1_ is the concentration of the ultrafiltration; C_2_ is the concentration of the original solution.

### 2.6. Antifouling Performance of Membranes

Antifouling performance of PVDF composite membranes was evaluated using the pure water flux recovery rate. The BSA solution (1 g/L) was selected as the test solution. Pure water flux was J_0_ and BSA solution flux was J_1_. The specific test process was as follows: the flux of pure water and BSA solution was measured once every 5 min within 120 min. After measuring the BSA solution, the membrane was cleaned with deionized water, and the pure water flux (J_2_) was measured again at 0.1 MPa. The flux recovery ratio (R_t_), irreversible fouling ratio (R_r_), reversible fouling ratio (R_ir_), as well as irreversible fouling ratio was calculated in Equations (3)–(6):

(3) $$\mathrm{FRR}=\frac{J_{2}}{J_{0}}\times 100\%$$

(4) $$R_{r}=\frac{\left(J_{2}-J_{1}\right)}{J_{0}}\times 100\%$$

(5) $$R_{\mathrm{ir}}=\frac{\left(J_{0}-J_{2}\right)}{J_{0}}\times 100\%$$

R_t_ = R_r_ + R_ir_ (6)

## 3. Results and Discussion

### 3.1. Structure and Morphology of Self-Made TiO_2_

**Figure 2:**
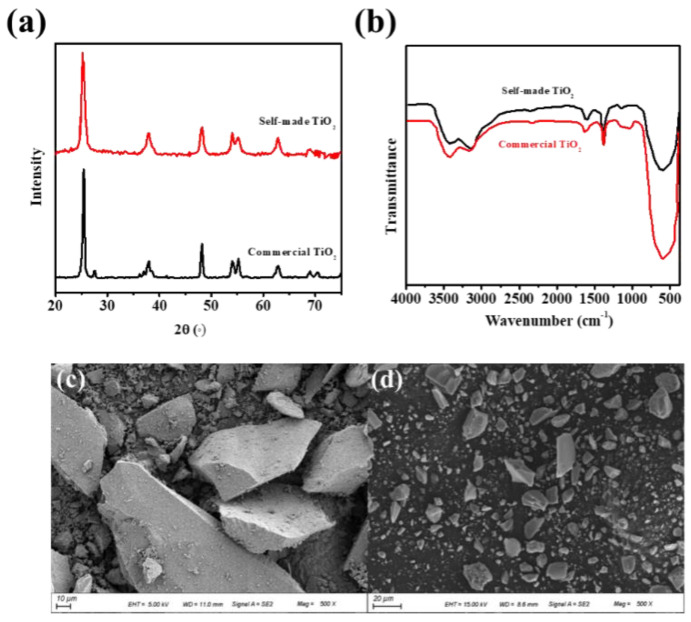
(**a**) XRD pattern of the self-made TiO_2_. (**b**) FT-IR spectra of commercial and self-made TiO_2_/PVDF composite membranes. (**c**,**d**) SEM picture of the self-made TiO_2_.

The crystal structure of the self-made TiO_2_ was studied using the X-ray diffraction (XRD) measurements. Figure 2a shows the XRD pattern of the sample. The crystallization of the self-made TiO_2_ allows to obtain a high anatase content and no rutile peaks were found. The anatase phase shows better properties due to higher electron mobility in the lattice of anatase compared to rutile. The chemical structure of commercial TiO_2_ and self-made TiO_2_ was investigated by Fourier transform infrared (FT-IR) spectrometry, as shown in Figure 2b. Compared with commercial TiO_2_, the peaks of -COOH at 1680 originating from the C=O from -COOH for self-made TiO_2_ were slightly stronger, indicating the increased hydrophilicity. Moreover, the high-magnification SEM image of the self-made TiO_2_ also clearly exhibited the smooth surface (Figure 2c,d), indicating the high hydrophilicity. Moreover, the contact angle measurements were further performed. The contact angle is responsible for membrane hydrophilicity. The hydrophilicity of self-made TiO_2_ can be seen (48.63°), in which the water contact angle decreases compared with the commercial TiO_2_ (50.8°). We further analyzed the water contact angle of PVDF composite membrane prepared with the commercial TiO_2_ and the self-made TiO_2_, respectively. Notably, the water contact angle of the PVDF composite membrane prepared with the self-made TiO_2_ was lower than that of the PVDF composite membrane prepared with the commercial TiO_2_, indicating the better hydrophilic properties.

The relatively higher hydrophilicity was found for self-made TiO_2_ due to the presence of hydroxyl functional groups on the TiO_2_ nanoparticle surface, which was consistent with the results of FT-IR.

### 3.2. Characterization of Composite Membranes

#### 3.2.1. Water Flux, Contact Angle, and Porosity

For the basic properties of the PVDF composite membranes modified by the particles altered with different content (Table 2), the pure water flux of the composite membranes increased after adding TiO_2_ particles, and the PVDF-2 composite membranes achieved the best pure water flux (288.3 L·m^−^^2^·h^−1^). The contact angle of the composite membranes decreased after modification with TiO_2_, which indicates that TiO_2_ can effectively improve the hydrophilicity of the membranes. Notably, the PVDF-2 has the lowest contact angle and porosity, and the highest BSA rejection rate. This phenomenon may be due to the increase in the viscosity of the casting solution as the self-made TiO_2_ was added into the PVDF casting solution. The mass transfer resistance of solvent and solute also increased during preparation of the gel, resulting in a slight decrease in water flux and porosity. Compared with the PVDF-1 membranes, PVDF-2 membranes showed a lower contact angle and higher BSA rejection rate, which could be attributed to the higher hydrophilicity.

#### 3.2.2. Phase Separation Rate

**Figure 3:**
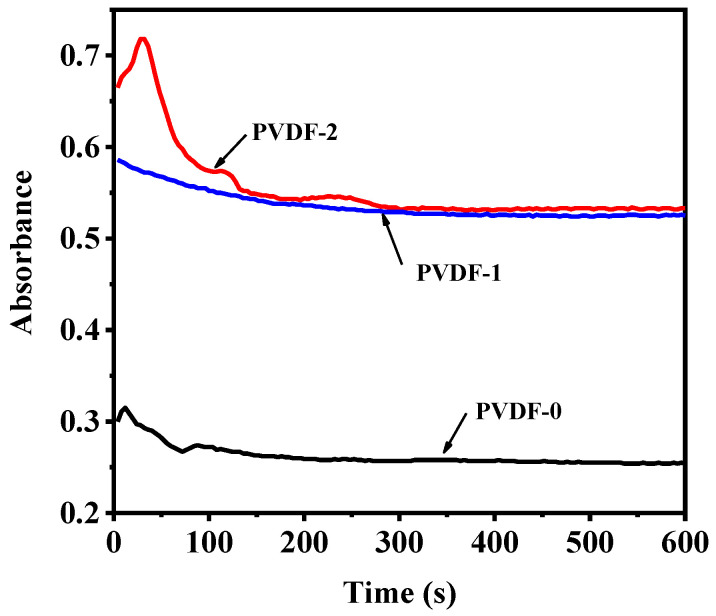
The absorbance change of DMF in deionized water of blank and composite membranes.

The phase separation rate and pore structure are affected by the mass transfer process. The phase separation velocity of casting solution in the process of membrane forming was analyzed by studying the absorbance change of DMF in deionized water. As shown in Figure 3, the absorbance of DMF in the casting solution with particles is greater than that of the blank casting solution. The results show that TiO_2_ particles accelerate the phase separation speed, which is due to the barrier of nanoparticles reducing the interaction force between polymer and solvent. Thus, the solvent molecules were more easily diffused from polymer matrix. In addition, the phase separation rate of PVDF membrane prepared by adding self-made TiO_2_ particles is higher than that of commercial TiO_2_ particles, which indicates that the self-made TiO_2_ particles are more conducive to the diffusion of solvent into water. The high phase separation speed indicates that the exchange rate between solvent and non-solvent is high, which was conducive to the formation of porous structure of the membrane, thus improving the filtration performance.

#### 3.2.3. Scanning Electron Microscopy

**Figure 4:**
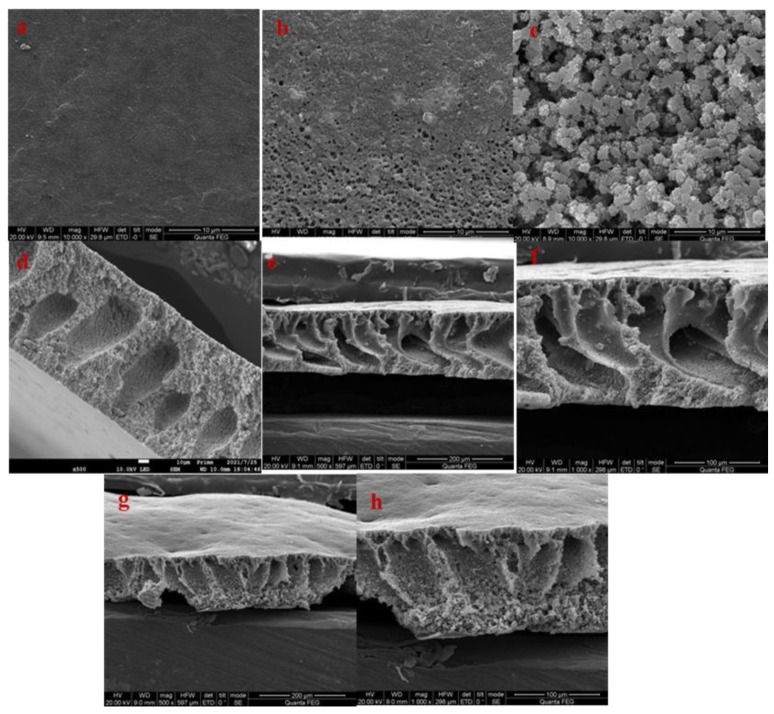
SEM pictures of (**a**) PVDF-0, (**b**) PVDF-1, and (**c**) PVDF-2. Section of (**d**) PVDF-0, (**e**,**f**) PVDF-1, and (**g**,**h**) PVDF-2.

Figure 4 shows the SEM results for the blank membrane and the composite membrane modified with commercial and self-made TiO_2_. Notably, the self-made TiO_2_ was evenly distributed in the PVDF. Compared with the blank membrane, the cross sections of the composite membrane were dense, indicating a porous structure. Clearly, the PVDF-2 achieves a denser and more uniform pore distribution on its surface. This is because the addition of self-made TiO_2_ can increase the mass transfer rate of the solvent and non-solvent in the process of membrane formation, which is conducive to the formation of pores and increases the roughness of the membrane surface, thus effectively improving the water flux of the membrane. The above SEM images show that the pore channels of the PVDF-2 are the most conducive to improving the fouling resistance of the membranes.

#### 3.2.4. FTIR Spectroscopy

**Figure 5:**
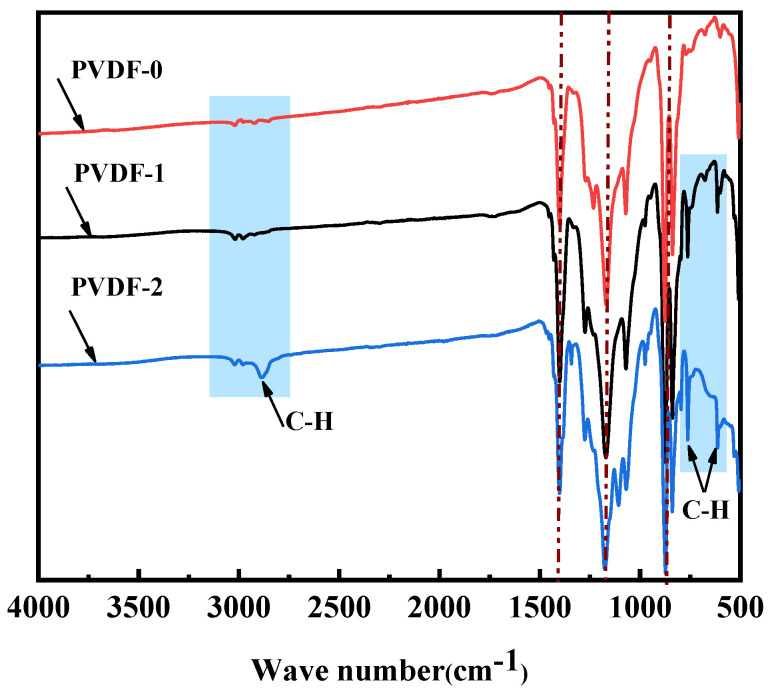
FTIR spectroscopy of the PVDF-0, PVDF-1, and PVDF-2.

The phases of the prepared composite membranes were analyzed using FTIR and the results are shown in Figure 5. The peaks at 1231, 834, and 525 cm^−1^ corroborate the γ phase of PVDF. The peaks at 1404, 1170, and 1071 cm^−1^ correspond to the C–F deformation. The phases of pure PVDF crystallized in the γ phase remained the same even after addition of modified TiO_2_ nanoparticles. Notably, a new peak at 2900 cm^−1^ appeared in the PVDF-2 membranes, which could be attributed to the hydroxyl groups, implying that the use of self-made TiO_2_ can improve the hydrophilicity of blank membranes.

#### 3.2.5. TGA

**Figure 6:**
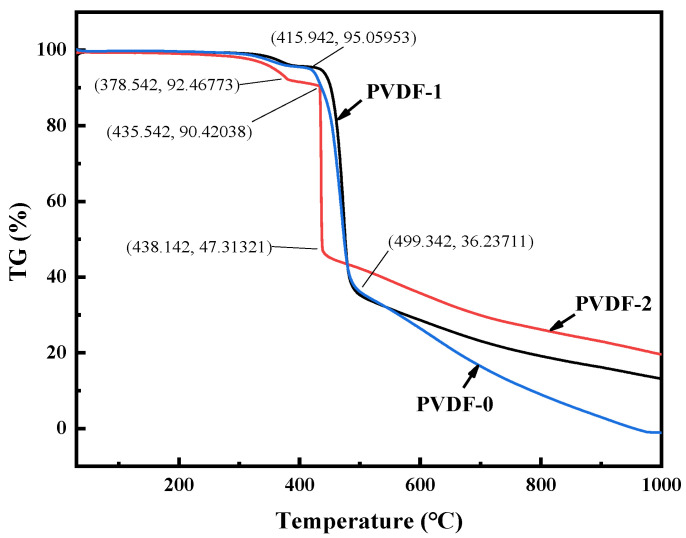
TGA of the PVDF-0, PVDF-1, and PVDF-1.

Figure 6 shows that the pyrolysis process of the PVDF changed after adding TiO_2_. Clearly, the decomposition temperature of the PVDF-2 (378.5 °C) is lower than that of the blank membrane (415.9 °C). This phenomenon may indicate the decomposition of functional groups on the surface of the modified membrane. Notably, the PVDF-2 showed the lowest decomposition temperature. The results show that the added self-made TiO_2_ becomes a nucleation center in the process of the PVDF solution-induced phase conversion, which affects the grain size and the void size of the PVDF formed in the process of film formation.

#### 3.2.6. Antifouling Performance

**Figure 7:**
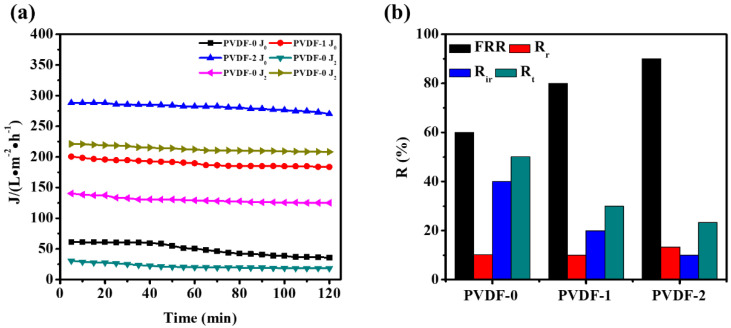
(**a**) The pure water flux of PVDF-0, PVDF-1, and PVDF-1. (**b**) The R_r_, R_ir_, R_t_, and FRR of PVDF-0, PVDF-1, and PVDF-1.

From Figure 7a, the pure water flux of PVDF membrane prepared by adding particles was greatly improved. Compared with the PVDF-0, the pure water flux of PVDF-2 increased to 288.3 L·m^−2^·h^−1^ and bovine serum albumin flux also increased 221.2 L·m^−2^·h^−1^. Notably, the flux of PVDF-2 did not change with time, indicating that the self-made TiO_2_ particles can effectively improve the pore structure and pores of the membrane.

Fouling analysis was undertaken by calculating the R_r_, R_ir_, R_t_, and FRR of PVDF-0, PVDF-1, and PVDF-2. These parameters are shown in Figure 7b. The flux recovery rate of PVDF-2 was 90%, which was more than 30% higher than that of the blank membrane. A higher FRR shows a better flux recovery while a lower R_ir_ demonstrates a better performance controlling the total fouling. The antifouling performance of the composite membrane was improved. The R_t_ values of PVDF-1 and PVDF-2 composite membranes were 30% and 23.3%, respectively, which were lower than those of pure PVDF membrane (50.1%). Moreover, the corresponding R_r_ and R_ir_ were also reduced. This may be because the stronger hydrophilic membrane was conducive to the formation of the hydration layer, thus reducing the contact between the membrane and pollutants to enhance the antifouling performance of the PVDF-2 composite membrane. The stronger antifouling ability of the composite membrane may be because the self-made TiO_2_ carries more negative charges, which increases the electrostatic repulsion to the pollutants and reduces the adsorption of pollutants on the membrane.

### 3.3. A/O-MBR Treatment for Landfill Leachate

The MBR system is composed of a first-class A/O and ultrafiltration membrane system. The ultrafiltration membrane system uses a built-in ultrafiltration membrane [39]. The aerobic tank in the activated sludge system is aerated by a blast, which can increase the oxygen utilization rate and agitation mixing ability of the system. Sampling is used to remove the excess sludge in the system. The traditional ultrafiltration membrane has poor anti-fouling and is plugged easily. However, the cleaning and preparation of the membrane has a high cost, indicating that the 3% TiO_2_ modified ultrafiltration processing is feasible for the MBR system [40].

#### 3.3.1. The Chemical Oxygen Demand (COD) Removal Characteristics of MBR

**Figure 8:**
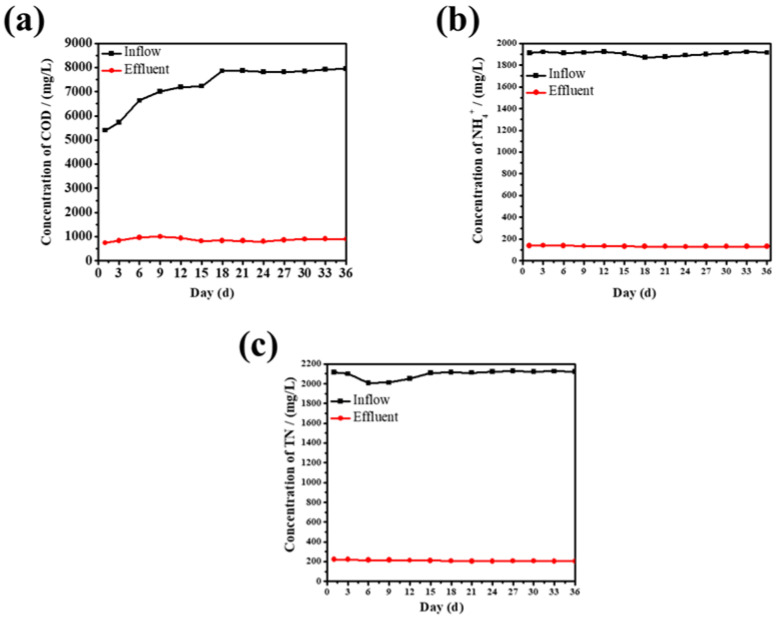
(**a**) Removal of COD; (**b**) removal of ammonia nitrogen; (**c**) removal of TN.

It can be seen from Figure 8a that during the operation of the system, the variation in COD in the influent is relatively large, ranging from 5000 to 8000 mg/L, which may be caused by the water quality characteristics of the leachate itself. However, the COD in the effluent of the system is relatively stable. The average COD removal rate of the system can reach 88%. It can be seen that the system features a good removal effect for COD and has a strong anti-impact load capacity that maintains a relatively stable removal rate. Ultrafiltration membranes play an important role in the system under a high organic load, which stabilizes and strengthens the quality of the leachate effluent and stabilizes the COD of the leachate effluent. In addition, the remaining 800 mg/L of the COD may be unable to be removed because most of the non-biodegradable and insoluble substances have also not been effectively removed.

#### 3.3.2. The Removal of Ammonia Nitrogen and Total Nitrogen by MBR

From Figure 8b,c, it can be seen that the influent of ammonia nitrogen and total nitrogen is relatively stable during the operation of the system. The influent concentration of ammonia nitrogen is about 1900 mg/L, the removal rate of the ammonia nitrogen is up to 93.2%, and the concentration of the ammonia nitrogen in the effluent is 130 mg/L. The concentration of the total nitrogen is about 2000 mg/L, the removal rate of the total nitrogen reaches 90.5%, and the ammonia nitrogen in the effluent is about 200 mg/L. In addition, the trends for the removal rates of the ammonia nitrogen and total nitrogen are basically the same, which may be due to the system still being in operation at the beginning. The microorganisms were not yet in their growth stage. However, with the passage of time, the microorganisms began to show activity, and the removal rate began to rise gradually. In addition, the total nitrogen decreased slightly at the beginning of the process, likely due to the relatively high influent COD concentration. The reduction in the total nitrogen mainly reflects the removal of ammonia nitrogen, which occurs because, under the same conditions, nitrifying bacteria will preferentially use dissolved oxygen, so the total nitrogen will decrease slightly in the case of a high water load and insufficient dissolved oxygen in the biochemical system. The ammonia nitrogen will then increase slightly. The increase in the ammonia nitrogen concentration inhibits the degradation of COD and nitrate nitrogen by aerobic bacteria.

#### 3.3.3. The Effect of Influential Factors on the Removal Efficiency of the Process

##### The Effect of Dissolved Oxygen (DO) on the COD, NH_4_^+^, and TN Removal

The amount of molecular oxygen dissolved in each liter of water determines the ability to deal with pollutants [41]. The nitrifying tank needs sufficient dissolved oxygen to promote the metabolism of nitrifying bacteria and other aerobic microorganisms. The dissolved oxygen in the denitrifying tank must be strictly controlled to adapt to the anaerobic respiration of anaerobic bacteria, such as denitrifying bacteria and facultative anaerobic bacteria. The pH of the MBR system was 7–8, the MLSS was 3200 mg/L, the HRT was 4 h, and the DO was 0–0.5 mg/L in the denitrification tank.

**Figure 9:**
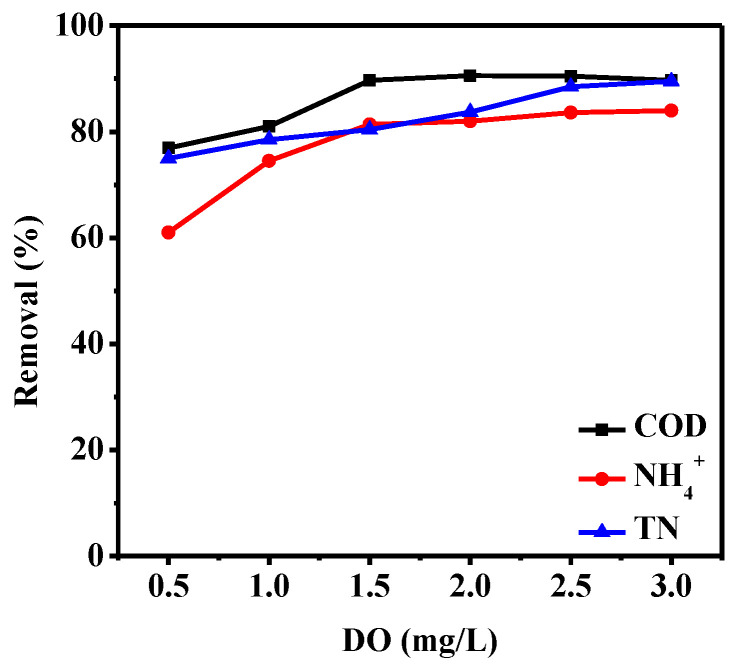
Effect of dissolved oxygen on COD, NH_4_^+^, and TN removal.

Figure 9 shows the variation curve of the COD effluent concentration with dissolved oxygen. As shown in Figure 9, the COD removal efficiency of the system is low when the concentration of DO is between 0.5 and 1 mg/L. This phenomenon can be attributed to the lower amount of DO in the system affecting the activity of aerobic bacteria and inhibiting aerobic bacteria. When the DO is between 1 and 1.5 mg/L, the COD removal efficiency of the system increases gradually from 80% to 89%. This occurs because an increase in DO is conducive to the growth of aerobic bacteria and the COD system. When the DO is between 1.5 and 2.5 mg/L, the MBR has the best COD removal effect, and the COD removal rate is about 90%. However, when the DO is 3 mg/L, the removal rate of COD by the MBR decreases, which may be due to the high aeration volume, the dispersal of activated sludge colloids, and the system’s reduced ability to treat organic matter. However, excessive DO has no effect on the refractory organic matter in the system, so the removal rate of COD does not rise but instead decreases.

As shown in Figure 9, the removal rate of ammonia nitrogen varies widely because nitrifying bacteria grow slowly, and the conditions of their growing environments are strict. The most important factor is that nitrifying bacteria are mainly located in the floc of activated sludge, and their biomass is relatively small [42]. Therefore, it is necessary to select an appropriate aeration intensity in the process of the experiment to make nitrifying bacteria more active in order to increase their activity. It can be seen from Figure 9 that when the dissolved oxygen in the nitrification tank is 0.5 mg/L, the removal efficiency of the ammonia nitrogen is the worst at 61%. This occurs because the final electron acceptor of nitrification is dissolved oxygen. When the dissolved oxygen content in the system is low, the activity of the nitrifying bacteria will be inhibited, and the conversion of ammonia nitrogen to nitrites and nitrates will be affected, resulting in a reduction in the ammonia nitrogen removal rate of the system [43]. When the dissolved oxygen in the nitrification tank is 1–3 mg/L, the removal efficiency of the ammonia nitrogen in the system increases gradually, and the highest removal rate can reach 90.56%. Thus, the ability of the system to remove ammonia nitrogen is closely related to dissolved oxygen. Xu Qiang et al. showed that when DO reaches a certain concentration and continues to increase the DO concentration, the efficiency of nitrogen removal in a reactor is not significantly improved. This is consistent with our research results.

In the A/O-MBR process, the removal of total nitrogen mainly depends on the biological denitrification process in activated sludge [44]. The key to biological denitrification is a smooth process of denitrification. The direct result of this successful process is the system’s removal of total nitrogen. In addition, the conditions of denitrification are anaerobic and anoxic, so it is necessary to strictly control the dissolved oxygen concentration in the reactor. Only under anoxic conditions can the denitrifying bacteria in the system denitrify with nitrate nitrogen as the electron acceptor and convert nitrate nitrogen into nitrogen, thereby reducing the total nitrogen of the system [45]. If the dissolved oxygen content is too high, the denitrifying bacteria can more easily obtain energy through aerobic respiration. During the experiment, the denitrifying tank was kept in a hypoxic environment of 0–0.5 mg/L.

Figure 9 shows that when the DO is 0.5–1 mg/L, the removal rate of total nitrogen is the lowest, with an average removal rate of only 76%. This occurs because the total nitrogen includes ammonia nitrogen and nitrate, and most nitrates are converted into nitrogen in a denitrification tank. When DO content is low, nitrification in an aerobic tank is inhibited, nitrite and nitrate production is relatively low, and the total nitrogen in the system is also relatively low. The removal efficiency decreases as the content increases. When the dissolved oxygen is 1.5–3 mg/L, the nitrogen removal capacity of the system gradually increases to 89.5%, at which point the nitrogen removal capacity of the system is the best. Thus, the removal of total nitrogen in the system occurs due to aeration blowing; on the other hand, denitrifying bacteria are dominated by nitrifying bacteria.

##### The Effect of pH on MBR Treatment COD, NH_4_^+^, and TN Removal

The basic metabolism of microorganisms in the process and living environment of pollutant degradation have strict requirements for pH [46]. Microorganisms have high activity in the range of pH 6–9. The metabolism of microorganisms beyond this range and the removal of pollutants will thus be inhibited at the same time. HRT = 4 h, MLSS = 3200 mg/L, DO = 1.5~2.5 mg/L, and DO = 0~0.5 mg/L in the denitrification tank during the experimental stage.

**Figure 10:**
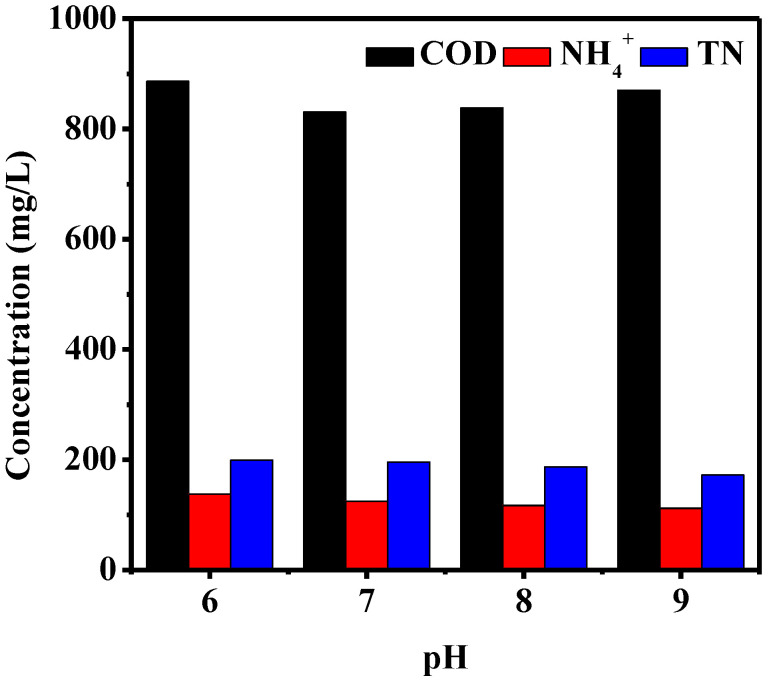
The effect of pH on COD, NH_4_^+^, and TN.

Figure 10 shows the COD of MBR effluent at different influent pH levels. When pH = 7, the COD of the effluent is the lowest; the average COD of the effluent is 830.67 mg/L, and the removal rate can reach 88%. When pH = 8, the average COD of the effluent is 838.17 mg/L. When pH = 9, the average COD effluent of the system is 870 mg/L. When the pH is 6, the average COD effluent of the system is 886 mg/L. Overall, when pH = 7~8, the microorganisms in the reaction pool can maintain a high activity. This system thus has a good biodegradation effect on organic matter. If the pH is too high or too low, biodegradation will be inhibited, which will affect the degradation of organic matter.

Figure 10 shows different pH conditions. The MBR system has the best effect on the removal of ammonia nitrogen and total nitrogen in the effluent when the influent pH = 8. The average concentration of ammonia nitrogen in the effluent is 111.67 mg/L, and the removal rate can reach up to 94%. The average concentration of total nitrogen in the effluent is 172.17 mg/L, and the removal rate reaches 92%. In addition to being degraded by microorganisms in the MBR, ammonia nitrogen can also be removed by aeration and partly converted into nitrate nitrogen and nitrite nitrogen in the effluent. When the system is at pH = 8.0, the removal efficiency of the NH_3_-N in the nitrification tank is the best, that of the nitrate nitrogen and nitrite nitrogen in the denitrification tank is the best, and that of the total nitrogen in system is also the best. When the influent pH was 9, the treatment of ammonia nitrogen and total nitrogen decreased slightly. The average effluent of ammonia nitrogen was 117.17 mg/L, the removal rate was 93%, and the average effluent of total nitrogen was 187 mg/L, while the removal rate was 91%. When pH = 7, the treatment effect of ammonia nitrogen in the system decreased with pH = 8. The average effluent of ammonia nitrogen was 125 mg/L, the removal rate was 89%, the total nitrogen effluent was 196 mg/L, and the removal rate was 90%. When pH = 6, the ammonia nitrogen effluent fluctuates greatly, the average effluent concentration is 137.5 mg/L, and the total nitrogen effluent is 199.17 mg/L. The graph shows that the removal of ammonia nitrogen by MBR mainly occurs due to the nitrification of the MBR biochemical system. When the nitrification activity of the nitrifying bacteria is the strongest, the appropriate pH is 8–8.4. Therefore, pH exerts the strongest effect on the removal of ammonia nitrogen in wastewater by influencing the activity of microorganisms in the biochemical system. MBR is the main agent that removes total nitrogen from nitrification and denitrification in the biochemical system, mainly through the filtration of the ultrafiltration membrane and the biological action of the microorganisms attached to the surface [47,48].

The metabolism of heterotrophic bacteria and the nitrification of nitrifying bacteria in the activated sludge will consume significant basicity during the operation of the MBR system, resulting in a decrease in the pH in the reaction. An excessively low pH will inhibit the removal of ammonia nitrogen by the bacteria. Therefore, when the MBR reactor is used to remove landfill leachate, the alkalinity of the leachate itself and the addition of alkaline substances should be used to adjust the pH of the reactor to achieve a higher ammonia nitrogen removal effect [49].

##### The Effect of Different Hydraulic Residence Times on MBR on COD, NH_4_^+^, and TN Removal Efficiency

Conventional landfill leachate treatment systems have long hydraulic retention times, which will require larger treatment facilities. The MBR can combine biochemical systems with ultrafiltration membranes. Ultrafiltration membranes can be used as secondary sedimentation tanks, thereby reducing the system’s footprint and the engineering budget [50,51,52,53]. The pH of the MBR system is 7–8, the MLSS is 3200 mg/L, the DO of the nitrification tank is 1.5–2.5 mg/L, and the DO of the denitrification tank is 0–0.5 mg/L.

**Figure 11:**
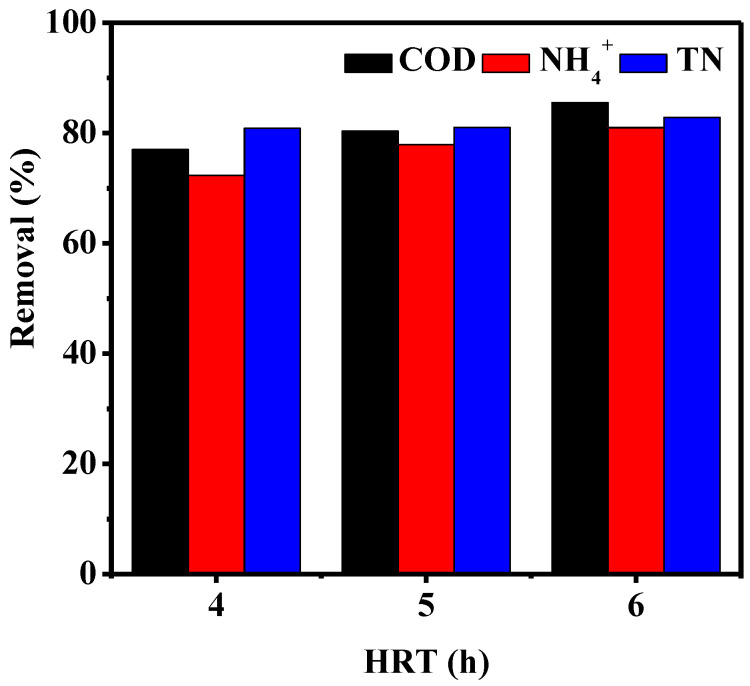
The effect of the hydraulic residence time on COD, NH_4_^+^, and TN removal.

As shown in Figure 11, hydraulic residence time is proportional to the COD removal rate. When HRT = 4 h, the removal rate of COD by the MBR system was 73%~85%, and the effluent effect was superior. When HRT = 5 h, the removal rate of COD in the MBR system tended to be stable—mainly between 76% and 86%. When HRT = 6 h, the removal rate of COD in the MBR system was 76.5~91%. Generally speaking, HRT has little effect on the COD removal rate because the MBR biochemical system can quickly decompose dissolved organic matter under an appropriate environment. However, the difficulty of organic matter degradation will not increase the possibility of degradation due to an increase in HRT.

Figure 11 shows that the hydraulic retention time is also proportional to the removal rate of ammonia nitrogen in the MBR system, with the highest removal rate being 89.5%.

As can be seen from Figure 11, with an increase in hydraulic retention time, the average removal rate of total nitrogen changed little (about 80%), possibly because, within a certain range, the hydraulic residence time of the system is positively correlated with the total nitrogen removal ability, but beyond a certain range, this residence time will lead to a large number of heterotrophic bacteria reproducing, thus inhibiting the activity of nitrifying bacteria, which is not conducive to denitrification, ultimately resulting in a low total nitrogen removal rate.

### 3.4. Water Outlet of RO

**Figure 12:**
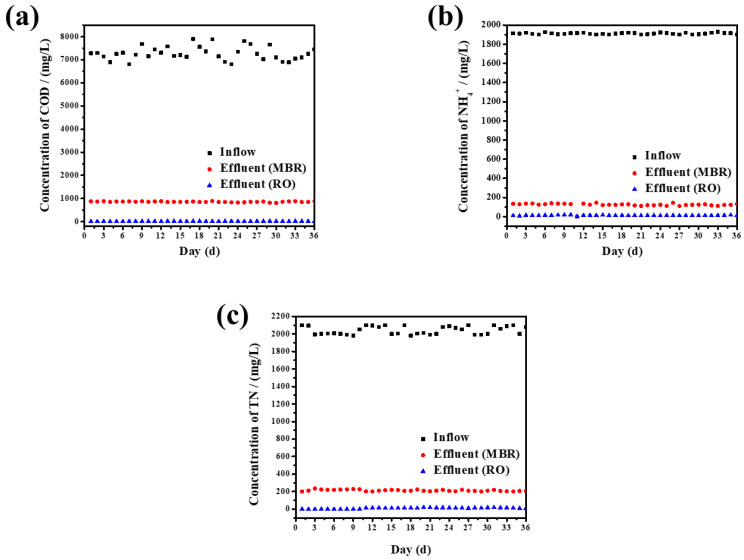
(**a**) Removal of COD; (**b**) removal of NH_4_^+^; (**c**) removal of TN.

Although the COD removal rate seems relatively high, the remaining COD concentration values (800 mg/L) are still high enough. The effluent of the MBR system enters into the RO system under the pressure of a special water pump. At the same time, it can desalt the effluent after MBR treatment, so as to ensure that the effluent quality after the subsequent treatment can reach the standard. The water quality of the effluent is shown in Figure 12.

## 4. Conclusions

PVDF membranes modified by TiO_2_ were prepared by the phase conversion method. The TiO_2_ nanoparticles were self-made and were more conducive to dispersion in the casting solution compared with commercial options. The introduction of TiO_2_ was conducive to the formation of pores and increased the roughness of the membrane surface, which effectively improved the water flux of the membrane. The water flux, porosity, contact angle, FTIR values, TGA values, and SEM pictures were measured. The results showed that the 5% self-made TiO_2_/PVDF had the best modification effect on the UF membrane, with a dense surface, good pore diameter, and a contact angle up to 63.21°. The A/O-MBR (modified membrane) was used to treat the garbage. In the leachate experiment, the average removal rates of COD, ammonia, and total nitrogen were 87.84%, 92.97%, and 89.95%, respectively. The best operating conditions for MBR are: MLSS = 3200 mg/L, nitrification tank DO = 1.5–2.5 mg/L, denitrification tank DO = 0–0.5 mg/L, pH = 7–8, and HRT = 5 h. To reach the standard, the effluent of the MBR system further enters into the RO system.

## Figures and Tables

**Table 1:** The parameters of membrane.

Name	Value
Inflow volume	10 L/h
Membrane flux	4.17 m^3^/h·dm^2^
Membrane element area	2.4 dm^3^
Working pressure	3~5 MPa
Clear liquid yield	80%

**Table 2:** Basic properties of the PVDF composite films modified by TiO_2_.

Sample Name	Pure Water Flux/L·m^−2^·h^−1^	Contact Angle/°	Porosity/%	R/%
PVDF-0	61.5	91.84	65.40	20.33
PVDF-1	200.6	84.22	70.58	42.36
PVDF-2	288.3	72.21	69.97	49.07

## Data Availability

Not applicable.

## Nomenclature

**Full Name**	**Abbreviation**
Anoxic–Aerobic Membrane Bioreactor	A/O-MBR
Upflow Anerobic Sludge Bed Membrane Bioreactor	UASB-MBR
Reverse osmosis	RO
Ultrafiltration membranes	UF
Titanium dioxide	TiO_2_
Sludge retention time	SRT
Membrane bioreactor	MBR
Poly(vinylidene fluoride)	PVDF
Hydrochloric acid	HCl
Rejection rate	R
Scanning electron microscopy	SEM
Fourier transform infrared spectroscopy	FT-IR
Thermogravimetric analysis	TGA
Chemical oxygen demand	COD
Ammonia nitrogen	NH_4_^+^
Total nitrogen	TN
Dissolved oxygen	DO
Mixed liquor suspended solids	MLSS
Hydraulic retention time	HRT
